# Refractory Splenic Marginal Zone Lymphoma Responsive to Combination Venetoclax and Bortezomib (Velcade) (V^2^) Therapy

**DOI:** 10.3390/curroncol29060328

**Published:** 2022-06-06

**Authors:** Kyle C. Roche, Peter A. DeRosa, Min-Ling Liu, Victor E. Nava, Anita Aggarwal

**Affiliations:** 1 Department of Medicine, George Washington University School of Medicine and Health Sciences, Washington, DC 20037, USA; kyleroche@email.gwu.edu (K.C.R.); anita.aggarwal@va.gov (A.A.); 2 Department of Pathology, The University of Maryland Medical Center, Baltimore, MD 21201, USA; 3 Department of Pathology, George Washington University School of Medicine and Health Sciences, Washington, DC 20037, USA; min-ling.liu@va.gov (M.-L.L.); victor.nava@va.gov (V.E.N.); 4 Department of Pathology, Veterans Health Administration Medical Center, Washington, DC 20422, USA

**Keywords:** splenic marginal zone lymphoma (SMZL), chemotherapy refractory, progression without transformation, relapsed/refractory (R/R), Venetoclax, Bortezomib (Velcade)

## Abstract

Standard treatment regimens for the management of patients with refractory splenic marginal zone lymphoma (SMZL) are currently unavailable. Here, we report a case of SMZL, which, after failing multiple therapeutics, demonstrated an impressive clinical response to combined Venetoclax and Velcade (V^2^), a treatment combination currently being investigated in the setting of refractory multiple myeloma. We also report a unique histopathology and mutational profile that may have important implications for the characterization and prognosis of SMZL.

## 1. Introduction

Splenic marginal zone lymphoma (SMZL) is a rare subtype of non-Hodgkin’s lymphoma that is thought to arise from memory B lymphocytes in the marginal zone of lymphoid follicles in the spleen [1]. Although it is usually a slow-growing tumor, on rare occasions it transforms into a high-grade lymphoma [2,3]. The rare and indolent nature of this disease has hampered efforts to establish a standard treatment regimen. Most data regarding treatment choice and efficacy have been derived from retrospective studies and unfortunately, randomized clinical trials are currently unavailable [1]. While watchful waiting is appropriate for asymptomatic patients, treatment is recommended for those who develop symptomatic splenomegaly, lymphadenopathy (LAD), or B symptoms. Treatment options include rituximab immunotherapy, splenectomy, chemotherapy, and combination therapies. Rituximab monotherapy and splenectomy are generally used as first line treatment options and demonstrate comparable survival rates in retrospective studies, noting that splenectomy is preferred for patients with large cell-transformation in splenic tissue or concurrent hepatitis C infection [1]. The addition of chemotherapeutics is generally reserved for patients with relapsed or refractory disease. Here, we present an unusual case of SMZL, along with a review of the literature.

## 2. Case Report

A 71-year-old African American male Vietnam Veteran presented with abdominal pain and dizziness in March of 2001. Computed tomographic scans (CT) showed hepatosplenomegaly with intra-abdominal LAD adjacent to the duodenum. Liver biopsy showed nodular lymphoid infiltrates composed of a mixture of small cells and scattered larger cells positive for CD20, kappa, BCL-2, IgD (focally), and BCL6 (mostly on few larger cells); and negative for lambda, CD5, CD10, and MUM1. The Ki67 proliferative rate was up to 15% and a few larger lymphoid cells were positive for P53. Peripheral blood-flow cytometry demonstrated surface kappa-restricted monoclonal B cells positive for CD20, CD19, CD22, and CD79b and negative for CD5, CD10, CD23, CD11c, CD25, and CD103 (Table 1). The overall findings were consistent with marginal zone lymphoma (MZL) of splenic origin.

The patient was lost for a follow-up until May of 2002, when he presented with post-prandial abdominal pain and 5–10 pounds of weight loss. Complete blood-count (CBC) revealed a white-blood-cell count (WBC) of 9.8 k/cmm (normal values: 3.2–9.5 k/cmm), hemoglobin (Hgb) of 12.6 g/dL (normal value: 11.2–15.6 g/dL), platelet count (Plt) of 180,000 k/cmm (152–375 k/cmm), and lactate dehydrogenase (LDH) of 294 U/L (normal values 135–225 U/L). Human immunodeficiency virus, hepatitis B, and hepatitis C were negative. Bone marrow (BM) biopsy showed hypercellularity for age, mainly due to erythroid and myeloid hyperplasia without left shift and small lymphoid aggregates (representing <10% of cellularity) with interspersed large lymphocytes, morphologically and immunophenotypically congruent lymphomatous involvement. CT scan revealed splenomegaly measuring 14 cm with an enlarged para-aortic lymph node (LN) (largest diameter of 4 cm). In September of 2002, after 4 weekly doses of rituximab monotherapy, CT monitoring revealed normalization of spleen size and some residual (sub-centimeter) retroperitoneal LAD. Notably, the Lugano classification and response assessment criteria were used as recommended by Cheson et al. [4].

**Figure 1:**
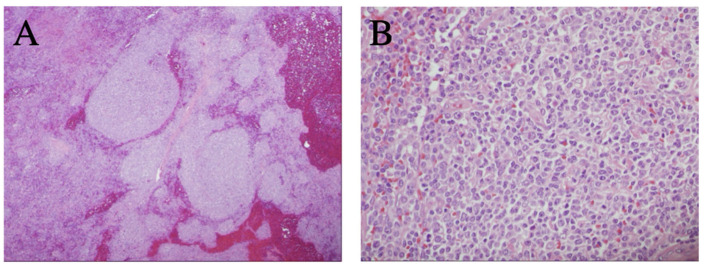
Representative histology observed in splenic tissue following splenectomy. (**A**) Expansion of splenic white pulp by marginal zone B-cell lymphoma without a classic targetoid pattern (H&E stain, 40×); (**B**) heterogenous lymphoma cells, including a mildly increased proportion of large/centroblastic cells and occasional mitoses, focally (H&E stain, 400×).

**Figure 2:**
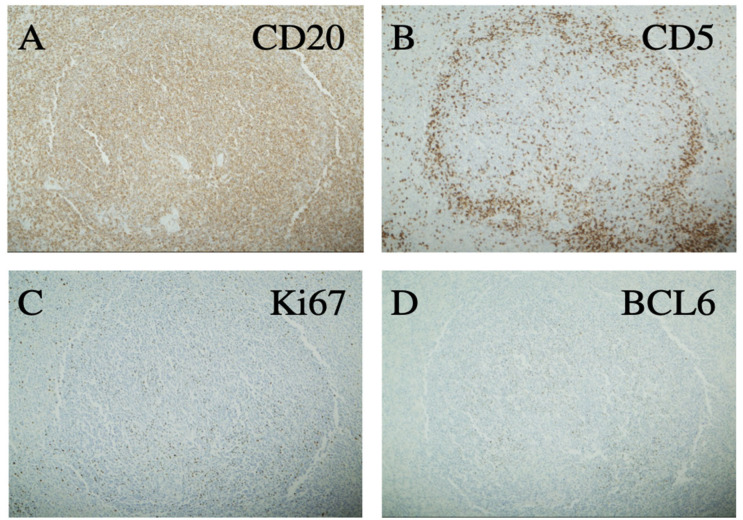
Immunohistochemistry of a representative splenic lymphoma nodule showing positivity for CD20 (**A**) and negativity for CD5 (**B**). Ki67 is focally increased above the usual level of low-grade B-cell lymphoma (**C**) and BCL6 is positive in a few larger cells without sheeting (**D**) All 100×.

As outlined in Table 1, the patient received multiple diagnostic and therapeutic surgical interventions over a course of 20 years (2001–2020), including BM and LN biopsies and splenectomy, which demonstrated stable morphology. Interestingly, the splenectomy specimen revealed lymphomatous infiltration of white and red pulp with a uniform nodular lymphomatous architecture (lacking distinguishable marginal zone/mantle zone interphase) (Figure 1) The specimen was also notable for increased scattered large cells that were variably positive for Bcl6 and P53 with a Ki67 proliferative rates up to ~20%, as previously seen in other samplings (Figure 2).

In December of 2011, a repeat BM biopsy showed increased lymphomatous burden (~50% of cellularity) without evidence of transformation. Karyotype analysis detected a new del (2) (q13q33) in addition to the complex karyotype previously observed: 45, XY, t (1; 17) (q25; q21), del (2) (q13q33), add (4) (p14), del (5) (q13q15) [4] (Table 1).

The patient received several courses of chemotherapy, including Rituximab monotherapy (4 weekly doses in 2002, 2004, 2007, 2010, 2012), splenectomy (2006), R-CVP (6 cycles in 2008), bendamustine and rituximab (2015), obinutuzumab and lenalidomide (2015–2016), ibrutinib alone initially, and then ibrutinib and rituximab (2016–2018). Note that some rituximab monotherapy courses were given per patient request at the time of disease progression.

In April of 2018, a CT scan showed stable hepatomegaly with worsening of LAD (largest 8 cm) in the mediastinum, abdomen, and pelvis. Positron emission tomography (PET) showed avid FDG uptake (Duvall score 5) in abdominal LNs and several areas of focal uptake in the liver, initially concerning for transformation into an aggressive lymphoma. However, a liver biopsy demonstrated known MZL with a mildly increased Ki67 proliferative index without evidence of transformation to diffuse large B-cell lymphoma (DLBCL). One course of Idelalisib therapy was given in July of 2018, with a follow-up CT demonstrating an initial 25% reduction in abdominal LAD (10/18). However, this regimen was discontinued due to the formation of a colonic abscess requiring colectomy.

In June of 2019, the patient complained of worsening abdominal pain and night sweats, and a CT scan revealed substantial hepatomegaly measuring 23 cm in greatest cranio-caudad dimension with stable LAD. A liver biopsy showed MZL again, with increased large cells and variable Ki67 proliferative rates up to ~20% in some areas without evidence of transformation. The patient was started on Venetoclax 50 mg PO in June of 2019, which was escalated to 400 mg PO without evidence of tumor lysis syndrome prior to adding weekly Velcade (V2). In October of 2019, a CT scan demonstrated a decrease in the size of intrathoracic, mesenteric, retroperitoneal, and deep pelvic LNs by 75–90%, as well as normalization of liver size when compared to prior studies. The patient reported subjective improvement in appetite, stabilization of weight, and no constitutional symptoms over subsequent follow-up visits. However, LDH values, which had been within normal limits, increased to 312 U/L in December of 2019. PET scan (01/20) demonstrated progression of previously identified lesions (increase by >50%) and numerous new lesions above and below the diaphragm, suggesting progression to a more aggressive lymphoma. 

A right axillary LN excision revealed known MZL with increased large cells without sheeting and a Ki67 proliferative rate focally increased to 30%. CD30 was negative and CD21/CD23 immunohistochemistry staining highlighted residual follicular dendritic meshworks ruling out transformation to DLBCL. Next-generation sequencing (NGS) of nodal tissue revealed the following mutations: CD36 I399fs*19, MYD88 L265P and V217F, MLL2 splice site 4237-57_4377del198, NOTCH2 R2400*, PHF6 M1K, TAF1 R843W (subclonal), TRAF3 rearrangement exon 10, and several variants of uncertain significance (ACTBM16I, ASXL1 N57S, BCORL1 P1007T, BRIP1 L56I, CD274 (PD-L1) splice site 52 + 2T > C, CEBPA R327W, CIC P660L, CREBBP F1484C, DNMT3A F751I, FLYWCH1 rearrangement, GNA13 Q27H, HIST1H1E L62V, HIST1H2BC E72K, MKI67 Q2084P, NTRK1 V211M, PCLO T2697A, PDCD1LG2 (PD-L2) C248fs*35).

At that time, treatment was discontinued. The patient died from SMZL in January of 2020 after an impressive clinical response for about 6 months with combination V^2^.

## 3. Discussion

Herein, a complicated case of SMZL in a 71-year-old African American male requiring multiple therapies during a 20-year prolonged course is presented. The patient’s clinical course was initially well controlled with multiple courses of rituximab monotherapy pre- and post-splenectomy. However, treatment resistance arose requiring additional intervention. Treatment with many therapeutics available for MZL achieved variable responses prior to disease progression to a more aggressive SMZL without transformation to DLBCL.

SMZL is a rare disorder, affecting 1.8 per 1,000,000 person years, and the indolent nature of SMZL has undercut efforts to establish a standard treatment regimen. Asymptomatic patients with SMZL can be under observation without treatment for many years with no change in overall outcomes [5,6]. Based on consensus guidelines, treatment for SMZL is only indicated for patients with symptomatic splenomegaly, progressive nodal disease, symptomatic cytopenias, and/or autoimmune cytopenias [7,8,9,10]. Rituximab therapy has become a first-line alternative to splenectomy, with outcomes that are comparable with or better than splenectomy 

Relapsed/refractory (R/R) patients with SMZL include those who failed local treatment (splenectomy) and those with disease progression following rituximab monotherapy. Retreatment of (R/R) patients with rituximab monotherapy is commonly employed as it retains its efficacy in most cases, however, consideration of rituximab-based chemotherapy regimens is also appropriate [11,12,13,14,15,16,17,18,19]. A few retrospective and prospective studies have assessed the utility of combination therapies to treat SMZL. Unfortunately, the combination therapy regimens differed considerably within and between studies, including various combinations of fludarabine, cyclophosphamide, cladribine, vincristine and prednisone, and liposomal daunorubicin [15,16,17,18]. Bendamustine-Rituximab has recently gained popularity as a preferred regimen in R/R SMZL based on the impressive ORR (92%) observed in the BRIGHT study in MZL patients [19].

Over the past 10 years, improvements in our ability to molecularly and genetically characterize MZL as it relates to underlying disease biology has led to the use of more targeted therapies with improved efficacy and tolerability. To this end, a few prospective studies investigating the use of targeted therapies for the treatment of MZL, including a small subset of SMZL, have shown promise.

Small molecule kinase inhibitors targeting BCR signaling, such as the Bruton’s tyrosine kinase (BTK) inhibitor (ibrutinib), seem to provide new avenues of therapeutic strategies [20,21,22,23,24]. A pivotal Phase II open-label international study of R/R MZL treated with single-agent ibrutinib reported an ORR of 48%, and the median PFS was 19.4 months for SMZL. Based on this study’s results, the FDA granted accelerated approval for ibrutinib for the treatment of R/R MZL requiring systemic therapy in patients with disease progression while receiving at least one prior anti-CD20-based therapy. A further final analysis of PCYC-1121 with median follow-up of 33.1 months (range: 1.4–44.6) demonstrated an ORR of 58%, a median duration of response (DOR) of 27.6 months (95% confidence interval [CI]: 12.1 to inestimable [NE]), and a median progression-free survival (PFS) of 15.7 months (95% CI: 12.2–30.4). A median overall survival (OS) was not reached (95% CI: NE to NE) [22].

The proteasome inhibitor bortezomib also has significant single-agent activity in R/R MZL with an ORR of 48% (CR 31%). Similar impressive results were noted when bortezomib was combined with rituximab in R/R MZL [25]. An ORR of up to 80% (with 54% CRs) was reported in patients receiving rituximab in combination with the immunomodulating agent lenalidomide, with no unexpected toxicities [26,27,28].

Velcade and venetoclax is a promising combination therapy for the management of refractory multiple myeloma [29]. Venetoclax as monotherapy has been shown to have an ORR of 34% in patients with R/R NHL. Subsequently, efforts have been focused on identifying additional therapeutic agents capable of potentiating the efficacy of venetoclax [30,31,32]. The treatment efficacy of Venetoclax is currently being evaluated in combination with bendamustine plus rituximab and in combination with obinutuzumab plus CHOP in patients with follicular lymphoma (phase II), with promising preliminary results [33,34]. Venetoclax dosing was increased to 400 mg daily using an established escalation protocol and velcade was added, given that the patient had already received all other therapeutic agents [35]. In the case presented above, we found a marked initial response to combination V^2^ with resolution of hepatomegaly and approximately three months of symptom-free survival, providing anecdotal support for this combination therapy in refractory SMZL or a subset with increased large cells.

Although some of the initial biopsies were limited in size, the histopathology (including the splenectomy specimen) was not classical for MZL, and best fits the so-called MZL with increased large cells [36], which has not been included in the current WHO lymphoma classification. The tumor cells represented a heterogeneous mixture of small mature lymphocytes and larger centroblastic cells in various proportions depending on the specimen. However, all cases showed a higher frequency of centroblastic cells than expected in a classical MZL, correlated with a focally increased Ki67 proliferative rate. For example, the spleen showed a macronodular pattern of infiltration with extensive involvement of the red pulp, instead of the typical micronodular pattern expected in low-grade SMZL. Furthermore, the mitotic rate and expression of Bcl6 and P53 were more prominent and more obvious in the last specimens, suggesting progression to intermediate grade disease or selective survival of more aggressive clones. Our case also may provide insight into how mutational status correlates with cancer progression. For instance, we found a presumably inactivating nonsense mutation in CD36, a gene that has been shown to be an independent favorable prognostic indicator in DLBCL [37]. In the setting of AML, CD36 expression was decreased in patients with relapsing disease refractory to chemotherapy when compared to the expression levels of CD36 present in patients at the time of diagnosis [38]. Further research is necessary to elucidate the role of this gene in oncogenesis and response to chemotherapy.

In summary, we report upon an impressive clinical response in a patient with refractory SMZL to combination venetoclax and velcade, which is currently being studied in the context of refractory multiple myeloma. This observation suggests that V^2^ therapy may represent an effective therapeutic option for refractory SMZL in the absence of clinical trials to guide treatment approaches.

## 4. Conclusions

The rare and indolent nature of SMZL has hampered efforts to discover effective treatment regimens, especially in patients with R/R disease. Clinical studies aimed at assessing the efficacy of various therapeutic combinations for the treatment of SMZL have proven useful; however, heterogeneity among treatment courses and limited sample sizes have limited our insight. Consequently, the discovery of novel treatment combinations for SMZL remains an area of active interest. We report upon an impressive clinical response in a patient with refractory SMZL to combination venetoclax and velcade (V^2^), which is currently being studied in the context of refractory multiple myeloma. This observation suggests that V^2^ therapy may represent an effective therapeutic option for refractory SMZL in the absence of clinical trials to guide treatment approaches.

## Figures and Tables

**Table 1:** Summary of tissue sample histology, immunohistochemical profile, and associated genetic mutations.

Date	Specimen	Pathology	Antigen Profile	Additional Characterization
1 February 2001	Liver Bx	Nodular lymphoid infiltrates composed of a mixture of small and scattered large cells	Positive: CD20, kappa, BCL2, focal IgD and BCL6 (mostly on few larger cells). Negative: Lambda, CD5, CD10 and MUM1	Ki67 < 15%; focal p53 expression (few large cells)
1 February 2001	Blood		Positive: CD20, CD19, CD22 and CD79b. Negative: CD5, CD10, CD23, CD11c, CD25, CD103	
1 June 2002	BM Bx	Hypercellular, nodular aggregates of small lymphocytes with interspersed large lymphocytes	Positive: CD20, kappa, BCL2, and BCL6 (mostly on few larger cells). Negative: Lambda, CD5, CD10 and MUM1	
1 April 2006	Spleen	Composed predominantly of small lymphocytes, some with plasmacytic differentiation and some large lymphs mainly in white pulp	Positive: CD20, CD19, CD22 and CD79b	Ki67: 15–20%; focal P53 expression (few large cells)
1 October 2007	BM Bx	Unchanged		Karyotype 46XY, del (2) (q13q33); t (1; 17), (q25; q21) add (4) (p14), del (2) (5) (q13q15)
1 September 2016	LAN Bx	Increased % of large cells	Unchanged	Ki67: up to 30%
1 May 2018	Liver Bx	Same as LN	Unchanged	Ki67: up to 30%
1 June 2019	BM Bx	Unchanged	Unchanged	Ki67: up to 30%
1 January 2020	LAN bx	Unchanged	Unchanged	Ki67: up to 30%

## Data Availability

The data presented in this study are available on request from the corresponding author.
